# Distinctive Anthocyanin Accumulation Responses to Temperature and Natural UV Radiation of Two Field-Grown *Vitis vinifera* L. Cultivars

**DOI:** 10.3390/molecules20022061

**Published:** 2015-01-27

**Authors:** Ana Fernandes de Oliveira, Luca Mercenaro, Alessandra Del Caro, Luca Pretti, Giovanni Nieddu

**Affiliations:** 1 Department of Agriculture, University of Sassari, Viale Italia 39, Sassari 07100, Italy; E-Mails: lucamercenaro@gmail.com (L.M.); delcaro@uniss.it (A.D.C.); gnieddu@uniss.it (G.N.); 2 Porto Conte Ricerche Srl, S.P. 55 Porto Conte/Capo Caccia, Tramariglio-Alghero (SS) 07041, Italy; E-Mail: pretti@portocontericerche.it

**Keywords:** anthocyanin accumulation and partitioning, UV radiation, temperatures, thermal time modeling, Mediterranean climate

## Abstract

The responses of two red grape varieties, Bovale Grande (syn. Carignan) and Cannonau (syn. Grenache), to temperature and natural UV radiation were studied in a three-years field experiment conducted in Sardinia (Italy), under Mediterranean climate conditions. Vines were covered with plastic films with different transmittances to UV radiation and compared to uncovered controls. Light intensity and spectral composition at the fruit zone were monitored and berry skin temperature was recorded from veraison. Total skin anthocyanin content (TSA) and composition indicated positive but inconsistent effects of natural UV light. Elevated temperatures induced alterations to a greater extent, decreasing TSA and increasing the degree of derivatives acylation. In Cannonau total soluble solids increases were not followed by increasing TSA as in Bovale Grande, due to both lower phenolic potential and higher sensitivity to permanence of high temperatures. Multi linear regression analysis tested the effects of different ranges of temperature as source of variation on anthocyanin accumulation patterns. To estimate the thermal time for anthocyanin accumulation, the use of normal heat hours model had benefit from the addition of predictor variables that take into account the permanence of high (>35 °C) and low (<15 °C and <17 °C) temperatures during ripening.

## 1. Introduction

There has been increasing international recognition of the interactions and feedback between climate change and surface UV radiation [1,2], but the understanding of such interactions and of the induced ecosystem changes are limited since they act over medium-long time scales [3,4]. These environmental issues have led to a growing interest of the scientific community in studying plant acclimation to both light and thermal effects of solar radiation, under natural or modified climate conditions [5,6,7,8,9,10,11].

In viticulture, particular interest has recently been given to the effects of high temperature and UV radiation on secondary metabolite accumulation, namely of flavonoids (anthocyanins and pro-anthocyanidins), amino acids and aroma compound precursors, for their fundamental importance to berry color formation and stability, and to wine flavor and astringency [10,12,13,14,15,16,17,18,19]. Together with genetics, plants nutritional and sanitary status, canopy architecture and density assume a major role on modeling plant adaptation to climate conditions [20,21,22,23,24]. The composition and concentration of anthocyanins and pro-anthocyanidins vary with the cultivar, cultural practices and microclimate conditions during berry development and ripening [11,13,25]. Though light interception at the cluster zone have a positive effect on berry skin anthocyanin accumulation [26], high sunlight exposure may cause a reduction on anthocyanin concentration, due to high temperatures exposure [24,27]. 

Several authors have demonstrated that a reduction on anthocyanin accumulation by high temperature (>30 °C) can result both from reduced synthesis and increased degradation of previously accumulated contents [12,14,28]. Furthermore, increased temperature after veraison may alter berry composition by reducing the anthocyanin: sugar ratio of ripe berries [29], probably due to a delay on anthocyanin accumulation, which shifts the onset of the linear phase in which anthocyanin and sugar increase in parallel [30]. Recent research works focusing on anthocyanin biosynthesis dependence on temperature and light [31] have demonstrated that light increases anthocyanin content in berry skin regardless of temperature and act synergistically with low temperatures (15 °C) on the expression of flavonoid biosynthetic-related genes.

As far as anthocyanin partitioning is concerned, light and temperature effects of solar radiation seem to influence differently anthocyanin accumulation and composition [31,32]. In Azuma* et al*. [31] experiment, low temperature and light affected anthocyanin composition of Pione (V. xLabruscana), increasing peonidin and malvidin derivatives while malvidin contents decreased in the absence of light and the two derivatives were reduced under high temperature conditions (>35 °C). High temperatures seem to alter anthocyanin composition also towards a higher acylation proportion of all derivatives [12,13,17]. On the other hand, berry sunlight interception seems to have a positive effect on dihydroxylated anthocyanin synthesis and a decreasing effect on trihydroxylated derivatives, as compared to complete shadow [33]. Inconsistent results have been observed regarding the effects of UV radiation on anthocyanin synthesis [12]. Nevertheless, recent works have observed a reduction of flavonols under UV-B filtering treatment [34,35] and an increase in berry skin anthocyanin content in response to UV-B radiation [36]. It seems that the accumulation of monosubstituted flavonols is increased upon UV-B light treatments [36,37]. Carbonell-Bejerano* et al*. [37] report enhanced petunidin acetylglucoside and delphinidin coumaroylglucoside levels in Tempranillo berry skin. Moreover, working with the same variety, Martinez-Lüscher* et al*. [36] have observed an increase in trisubtituted and methylated anthocyanins under UV-B light treatment and also increases the acetylation level, both with acetic and *p*-coumaric acids. In addition, distinctive varietal responses to light may be observed in flavonols and anthocyanin accumulation and composition in berry skin [32,33,38]. The aim of this study was to analyze the effects of natural UV light radiation and of the permanence of high and low temperatures on berry skin anthocyanin contents and composition of two grapevine varieties traditionally cultivated in western Sardinia (Italy), Bovale Grande (syn. Carignan) and Cannonau (syn. Grenache), with distinct phenolic potential [32,39,40]. Both varieties were subjected to reduced UV light (*i.e.* visible and visible + UV-A transmittance) under natural field-growing conditions. Along the three seasons of this experiment we analyzed ambient light intensity and spectral composition see [32], temperature and other variables derived from these, which are known to be basic weather variables affecting berry development and composition. We monitored berry skin temperature, from veraison until harvest, as well as berry skin total anthocyanin contents, and we used hierarchical linear regression to test the modeling effects of different ranges temperature as source of variation in anthocyanin accumulation patterns.

## 2. Results and Discussion

### 2.1. Experimental Season Thermal Conditions

In Table 1 the monthly average temperatures recorded in the study area during seasons 2009, 2010 and 2011, and the variation from the long term average, are reported. The first season was characterized by high UV intensities during the period from June to September [32,41] and by a much greater prevalence of high air temperatures (>30 °C), as compared both to the long term 30 year average and seasons 2010 and 2011 [32,41,42,43]. In 2009, the maximum temperature (T_max_) during berry growth and development reached a monthly average value of 27.5 °C in June which was about 3 °C higher than the 30 year average and the seasons 2010 and 2011. Again, in July T_max_ averaged 31.1 °C, nearly +3 °C than 2011 and long term average but about +1.5 °C higher compared to 2010.

The months of August and September 2009 continued recording temperatures higher than the 30 year average for the same period, while the seasons 2010 and 2011 were much less hot and the maximum temperature values remained close to those of long term data, or even lower than the average values (−0.7 and −0.3 °C during ripening 2010). Also mean (T_med_) and minimum (T_min_) temperatures registered much higher values than the average in 2009 (about +2.4 and +1.2 °C for T _med_ and +1.5 and 0.4 °C for T_min_ during ripening months). Conversely in 2010, a lower T_min_ was registered in August and September (−0.4 °C and −0.6 °C, respectively) and in 2011, T_min_ in August remained 0.6 °C below the average value, while in September it was only slightly higher than the average (about +0.3 °C).

**Table 1:** Monthly temperature conditions during the 2009‒11 growth seasons (from June to September) and long-term monthly 30-year average (1971 to 2000) in Capo Frasca, Italy [42,43]. Average values (x) and variation (Δ) between the study periods and the 30 year average.

Variable	Period	June		July		August		September	
		x	Δ	x	Δ	x	Δ	x	Δ
T_max_ (°C)	2009	27.5	3.0	31.1	3.3	30.3	1.5	26.9	0.9
	2010	24.6	0.1	29.3	1.5	28.1	−0.7	25.7	−0.3
	2011	24.9	0.4	27.6	−0.2	29.7	0.9	26.8	0.8
	30 year	24.5		27.8		28.8		26.0	
T_med_ (°C)	2009	24.5	3.6	27.5	3.5	27.3	2.4	23.5	1.2
	2010	22.1	1.2	26.4	2.4	25.3	0.4	22.6	0.3
	2011	22.2	1.3	24.4	0.4	25.7	0.8	23.7	1.4
	30 year	20.9		24.0		24.9		22.3	
T_min_ (°C)	2009	19.9	2.6	22.5	2.4	22.6	1.5	19.1	0.4
	2010	17.9	0.6	22.3	2.2	20.7	−0.4	18.1	−0.6
	2011	18.2	0.9	20.5	0.4	20.5	−0.6	19.0	0.3
	30 year	17.3		20.1		21.1		18.7	

Notes: T_max_, average maximum temperature; T_med_, mean temperature; T_min_, average minimum temperature.

### 2.2. Light Microclimate into the Fruit Zone

Under the plastic films, photosynthetically active radiation (PAR) intensity was attenuated to about 18% of the external ambient values. At the fruit zone, PAR attenuation at solar noon ranged on average from 90% to 98% of the ambient PAR values in the inner canopy layers, up to a minimum of about 51% and 67% in the external canopy layers of the Control treatments (Table 2). In the UV-screening treatments, UV-A and UV-B radiation intercepted by the clusters was reduced to about 10% and 30% of that measured in Control berries directly exposed to natural sunlight and no significant differences between canopy sides were observed at solar noon (Table 2).

**Table 2:** Photosynthetically active radiation (PAR) attenuation, UV-A and UV-B radiation intensity in East and West canopy sides, measured at solar noon in a clear sky day of veraison 2010, in Bovale Grande and Cannonau fruit zone. Mean values (*n* = 12) ± SE.

		PAR Attenuation (% Reference PAR)		UV-A (W·m^−2^)		UV-B (mW·m^−2^)	
Distance from the Canopy Centre (cm)		0–10	10–20	East	West	East	West
**Bovale Grande**	Control	90 ± 7.4	51 ± 9.6	3.0 ± 0.70	2.7 ± 0.55	143.7 ± 59.2	150.0 ± 56.5
	Vis + UV-A	92 ± 2.1	84 ± 2.4	0.8 ± 0.04	0.4 ± 0.04	13.1 ± 4.6	1.0 ± 0.1
	Vis	94 ± 3.5	81 ± 4.1	1.0 ± 0.10	0.3 ± 0.11	7.2 ± 2.3	3.0 ± 2.3
**Cannonau**	Control	90 ± 6.0	67 ± 12.3	1.5 ± 0.52	1.9 ± 0.61	130.0 ± 44.9	102.3 ± 53.8
	Vis + UV-A	98 ± 0.7	88 ± 2.1	0.6 ± 0.18	0.8 ± 0.13	5.2 ± 2.1	12.2 ± 8.5
	Vis	96 ± 1.2	88 ± 2.4	0.6 ± 0.17	0.7 ± 0.11	1.7 ± 0.8	4.5 ± 1.8

The light regimes induced significant differences in cluster light microclimate. PAR and UV radiation intercepted at cluster zone were significantly attenuated under the screening films, especially at midday due to the smaller solar angle and the shading effect of the canopy above. In the Control clusters UV-A and UV-B transmittances were significantly higher than that measured under the screening films, particularly during mid-morning (at 11.00 h) and beginning of the afternoon (at 15.00 h) [32]. The UV-A intensity reached similar values at the fruit zone in Vis + UV-A and Vis treatments but the UV-B transmittance and the ratio UV-B/UV-A were statistically lower in Vis all through the day. An extended characterization of cluster light microclimate during this experiment can be found in [32]. UV filtering films did not alter significantly the permanence of elevated temperatures (>35 °C) on berry skin, except for 2010, when Control berries were exposed to high temperatures for a longer period as compared to Vis + UV-A.

### 2.3. Berry Skin Temperature

**Figure 1:**
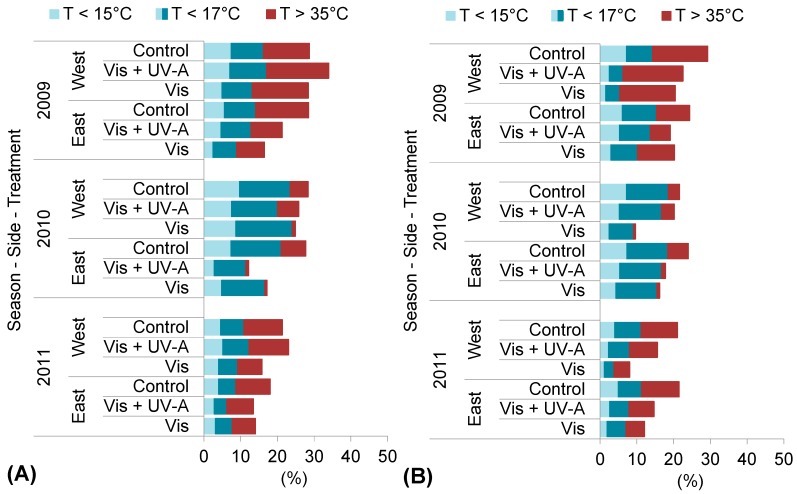
Berry skin exposure to defined ranges of temperature (<15 °C, <17 °C and >35 °C) in east and west canopy sides, during ripening in (**A**) Bovale Grande and (**B**) Cannonau.

Figure 1 reports the permanence of defined ranges of berry skin temperature (T_b_) during ripening. For each season, we calculated the 10th and 90th percentile of T_b_ in order to determine specific low and high temperatures (<15 °C, <17 °C and >35 °C) that, due to their frequency, could have affected berry skin metabolism.

Berries in the west side of the canopy were much more exposed to high temperatures than those in the east side. Furthermore, during ripening 2009, the prevalence of elevated temperatures (>35 °C) was significantly higher in all treatments and in both canopy sides as compared to the two following seasons. No variety effect was observed, and in Bovale Grande the permanence of such temperatures ranged from nearly 8% of the duration of ripening in East Vis and Vis + UV-A berries to 14% and 16% in Control and in West Vis and Vis + UV-A berries. In Cannonau, the results were similar, although the differences between east and west sides were higher in Control berries. In 2010, East Vis and Vis + UV-A berries were exposed to more than 35 °C for less than 1% of the entire ripening period in both varieties, while Control and West Vis + UV-A berry skin had more than 35 °C for about 6% of the time. In 2011, all treatments were subjected to more than 35 °C for a similar amount of time, the greatest difference being recorded in Cannonau between Vis and Control berries (from 5% to 10% of the time, respectively). The smallest prevalence of low temperatures (<15 °C) was observed in 2009, specially in Cannonau Vis berry skin (for about less than 2% of the duration of ripening). In that year, Control and Vis + UV-A berry skin reached less than 15 °C with higher frequency, lasting 5% and 7% of the time below this threshold in both varieties. In 2010, the percentage of time for which Control and Vis + UV-A berry skin remained with less than 15 °C was about 7% in Cannonau and 9% in Bovale Grande, and in 2011 it decreased to 4% and to 5% respectively.

Overall, the season 2011 showed the lowest permanence of T_b_ inferior to 17 °C and also the higher permanence of milder temperatures (ranging from 17 °C to 35 °C). This result is in accordance to the previously described air temperature conditions, since among the three seasons, the third was in fact the one in which air temperatures remained closer to the 30 year average.

### 2.4. Berry Skin Anthocyanins

The effect of light regime in berry skin anthocyanin content (TSA) at harvest was inconsistent and only statistically significant in Bovale Grande during the season 2010 and in Cannonau during 2009, when control berries were able to accumulate a significantly higher content of TSA as compared to the two UV-screening treatments (Table 3). No significant differences were observed between the two UV screening treatments but in the last two years of trial slightly higher mean values were observed in Cannonau Vis + UV-A. Also, Spayd* et al.* [12] obtained inconsistent results while Martínez-Lüscher* et al*. [36] have reported that, in Tempranillo berries, although higher concentrations of extractable anthocyanins had been observed, UV-B light did not alter total anthocyanin concentration. Azuma* et al.* [31] studies have demonstrated that high temperature (>35 °C) severely decrease TSA in berry skin and that low temperature (15 °C) and light induce anthocyanin accumulation in a synergetic manner. In our study both varieties were exposed to high temperatures for long time during 2009, with a small permanence of low temperatures (<17 °C). As compared to the previous year, in 2010, direct light exposure promoted higher anthocyanin accumulation in Control berries of Bovale Grande but not in Cannonau. In this variety, Vis + UV-A and Vis berries TSA concentration was probably enhanced due to the effect of lower permanence of high temperatures and higher permanence of low temperatures (Figure 1).

Cannonau showed significantly lower TSA contents as compared to Bovale Grande in all three seasons. Yet, for Cannonau Vis + UVA treatment, we observed an increase of about 80% in TSA both in 2010 and 2011 as compared to the hot 2009. The same pattern was observed for the Vis treatment: an increase in TSA of 230% in 2010 and 95% in 2011 for Vis. In Bovale Grande, the variation in TSA content between 2009 and the other two years of trial was more relevant in absolute value, but not in percent variations, since for both Vis + UVA and Vis treatments the variation ranged from +50% to +92%.

**Table 3:** Effects of light regime on total skin anthocyanin content (mg malvidin kg^−1^ berry) in Bovale Grande and Cannonau berries at harvest. Mean values (*n* = 9) and one-way ANOVA. Small letters indicate significant difference of mean values between treatments and ns refers to non-significant differences between treatment.

		Bovale Grande			Cannonau		
		2009	2010	2011	2009	2010	2011
**Treatment**	Control	306.9	585.8 ^a^	654.1	119.5 ^a^	127.0	102.1
	Vis + UV-A	298.8	450.3 ^b^	638.4	80.9 ^a,b^	182.1	143.2
	Vis	258.3	496.5 ^b^	650.2	55.5 ^b^	146.4	108.6
	Sig.	ns	< 0.05	ns	<0.05	ns	ns

Notes: Lower case letters in the same column indicate significant difference and ns refers to non-significant difference of mean values between treatment (*p* < 0.05) for each variety.

Though having important agronomic and oenological aptitudes [40,44], many accessions of Cannonau have shown low phenolic potential, namely regarding TSA. However, in sunny and warm climate conditions, using deficit irrigation strategies, this behavior can be partially compensated by an accumulation of higher proportion of more color stable forms of anthocyanins [45,46].

**Figure 2:**
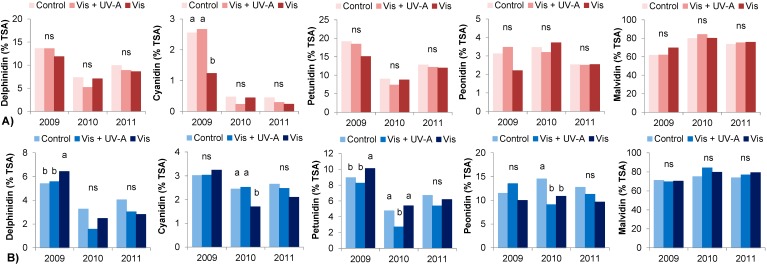
Proportion of TSA in delphinidin, cyanidin, petunidin, peonidin and malvidin based anthocyanins in Bovale Grande (**A**) and Cannonau (**B**) berries at harvest 2009, 2010 and 2011. Mean values (*n* = 9) and one-way ANOVA. Lower case letters (a and b) above the bars indicate significant difference and ns refers to non-significant difference of mean values between treatment (*p* < 0.05).

**Figure 3:**
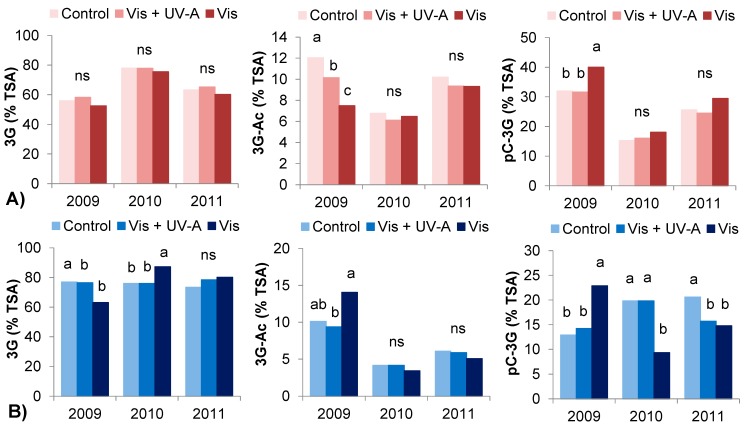
Proportion of TSA in 3-monoglucoside (3G), 3-acetyl-glucoside (3G-Ac) and 3-p-coumaroyl-glucoside (pC-3G) forms in Bovale Grande (**A**) and Cannonau (**B**) berries at harvest 2009, 2010 and 2011. Mean values (*n* = 9) and one-way ANOVA. Lower case letters (a and b) above the bars indicate significant difference and ns refers to non-significant difference of mean values between treatment (*p* < 0.05).

In our work, light regimes have influenced berry skin anthocyanin composition differently in the varieties and among seasons. In Figure 2 the proportion anthocyanin derivatives berry skin in Bovale Grande at harvest 2009, 2010 and 2011 are presented. In the hot season 2009, a higher proportion of cyanidin and peonidin glucosides was observed in Control and Vis + UV-A berries during ripening and at harvest, which is in accordance with previous studies suggesting a positive effect of light on dihydroxylated anthocyanins [33]. However, in the following years the treatments did not differ significantly in Bovale Grande and in Cannonau, the proportion of these derivatives was only significantly different between light treatments in 2010. Besides, the exposure to natural UV light intensities did not induce differences in trisubtituted anthocyanins in Bovale Grande and a decrease in these forms was observed on Cannonau Control berries during 2009. In 2009, Bovale Grande Control and Vis + UV-A berries presented higher proportion of acetylglucoside forms (Figure 3), probably due to both the combined effect of UV light and higher permanence of elevated temperatures [12,13,17,34,36]. In 2010 and 2011, the differences between treatments were not so evident. Yet, Cannonau Control berries presented higher acylation degree with coumaric acid, and higher contents of all anthocyanin derivatives at harvest, except for malvidin glucosides (Figure 3), which can be ascribed to a combined effect of the light treatment and a higher permanence of low temperatures in those years [31].

Major differences were observed among seasons (Table 4). In the two cultivar, the elevated temperatures of 2009 lead to higher accumulation of delphinidin and petunidin, and less peonidin and malvidin derivatives (Figure 2) in accordance to the results obtained by other authors [12,13,17]. In addition, in 2009 a very significant increase in the proportion of acylated forms was evident right from the beginning of ripening, with much lower monoglucoside contents in both varieties and in every light treatment (Figure 3, Table 4). At harvest 2011, the variation in the proportion of anthocyanin derivatives showed a trend similar to that observed in 2009, with significantly higher proportion of delphinidin and petunidin derivatives than in 2010 in Bovale Grande, especially in Control berries, and a much lower proportion of peonidin and malvidin in all treatments as compared to 2010.

These results are in accordance to those reported by Azuma* et al.* [31] who observed an increasing peonidin and malvidin derivatives under light and low temperatures. As far as the acylation degree is concerned, again, in 2011 berry skin anthocyanin profile showed an intermediate content as compared to the other two seasons, with significantly higher anthocyanin acylation than in 2010, probably due two higher permanence of elevated temperatures (Table 4). Light regimes affected anthocyanin partitioning in the two varieties, but the influence of natural UV light intensities on anthocyanin metabolism can be largely surpassed by that of high temperatures, both via anthocyanin degradation and increased acetylation. A detailed analysis on this issue can be found in [32].

**Table 4:** Effect of light treatment on berry skin percent composition of monoglucoside and acylated anthocyanins in Bovale Grande and Cannonau at harvest 2009, 2010 and 2011. Mean values (*n* = 9) and one-way ANOVA.

		Bovale Grande			Cannonau		
		Control	Vis + UV-A	Vis	Control	Vis + UV-A	Vis
**Monoglucosides**	**2009**	56.0 ^b^	58.2 ^b^	52.5 ^b^	77.0	76.4	63.1 ^b^
	**2010**	78.0 ^a^	77.8 ^a^	75.5 ^a^	76.0	76.0	87.3 ^a^
	**2011**	63.4 ^b^	65.2 ^b^	60.2 ^ab^	73.3	78.4	80.2 ^a^
	**Sig.**	<0.05	<0.05	<0.05	ns	ns	<0.05
**Acetylglucosides**	**2009**	12.1 ^a^	10.2 ^a^	7.5 ^b^	10.1 ^a^	9.4	14.0 ^a^
	**2010**	6.8 ^b^	6.1 ^b^	6.5 ^b^	4.2 ^b^	4.2	3.4 ^b^
	**2011**	10.2 ^a^	9.4 ^a^	9.3 ^a^	6.1 ^c^	5.9	5.1 ^b^
	**Sig.**	<0.05	<0.05	<0.05	<0.05	<0.05	<0.05
**Coumaroylglucosides**	**2009**	32.0	31.6 ^a^	40.0 ^a^	12.9 ^b^	14.2	22.9 ^a^
	**2010**	15.3	16.0 ^b^	18.1 ^b^	19.8 ^a^	19.8	9.3 ^c^
	**2011**	25.6	24.5 ^ab^	29.4 ^ab^	20.6 ^a^	15.7	14.8 ^b^
	**Sig.**	<0.05	<0.05	<0.05	<0.05	ns	0.05

Notes: Small letters in the same line indicate significant difference and ns refers to non-significant difference of mean values between treatment (*p* < 0.05) for each variety.

### 2.5. Thermal Efficiency for Berry Skin Anthocyanin Accumulation

**Figure 4:**
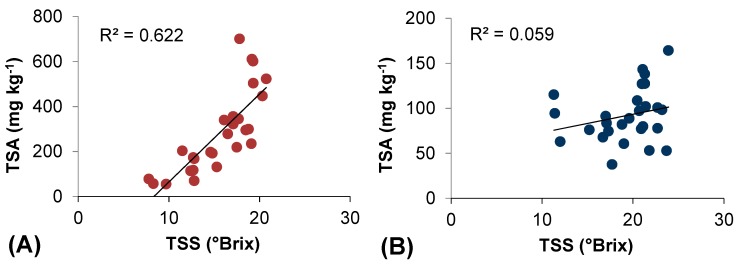
Relationships between total soluble solids (TSS) and total berry skin anthocyanin (TSA) content in (**A**) Bovale Grande and (**B**) Cannonau datasets.

Our results suggest that high and low temperatures were more effective than light treatment on influencing anthocyanin accumulation in Cannonau berry skin (Table 3, Figure 2 and Figure 3). Greater sensitivity to anthocyanin decrease driven by high temperature was observed in Cannonau [32]. Contrary to Bovale Grande and many other varieties [30], it is extremely difficult to observe the typical two-phase relationship between dynamic of anthocyanin accumulation and that of sugars (°Brix) in Cannonau. In our study, we plotted TSA with total soluble solid (TSS) data from the three years of experiment, and we obtained two completely different scatterplots for the two varieties (Figure 4). Bovale Grande showed a classic linear relationship between data [30], with a first lag phase where TSS increases and very small changes occur in TSA, followed by a nearly linear phase where both compounds increase in parallel. Conversely, Cannonau data does not fit any geometrical curve, but present a quite random dispersion of points, much evident under high temperature conditions. Besides for its genetically feeble phenolic potential, such behavior could partially be explained by a high sensitivity to the permanence of critical ranges of temperature [30,31]. In fact, as compared to warm and low altitude sites, in Mediterranean mountain terroirs, where weather conditions are characterized by higher daily temperature ranges, the pattern of berry skin anthocyanin accumulation is linearly correlated with TSS increments and considerably greater TSA contents have long been reported [46].

In order to better understand the effects of temperature on berry skin anthocyanin accumulation, we calculated the accumulated thermal time for anthocyanin synthesis, using the normal heat hours (NHH) model [47,48] and we determined the permanence (in hours) of low (<15 °C and <17 °C) and high (>35 °C) temperatures, based on berry skin temperature recorded during ripening. We then tested the relationships between TSA and the four predictor variables (NHH, H_T > 35 °C_, H_T < 15 °C_ and H_T < 17 °C_) for each variety.

Table 5 and Table 6 show the main regression analysis estimates and the model performances of three models tested for Bovale Grande and Cannonau, respectively. The first model estimates TSA contents based on a single variable, the NHH, which was statistically highly significant and showed a very good fitting for Bovale Grande, with correlation (R) and determination (R^2^) coefficients of 0.861 and 0.741, respectively. Conversely, despite being a highly significant variable in the simple linear regression for Cannonau model 1 (variable *p*-value = 0.004), the NHH was poorly correlated with TSA, with an R of 0.511 and a R^2^ of only 0.235. In both varieties, the introduction of the second variable H_T > 35 °C_ resulted in an improvement of the regression model, especially for Cannonau, with no collinearity problems between predictor and dependent variables, as indicated by the values of tolerance and variance inflation factor. Nevertheless, as far as Bovale Grande is concerned, the second model only resulted in a very slight increase of R and R^2^ as compared to model 1, and H_T > 35 °C_ *p*-value was indicative of non-statistical significant contribution of this variable for the overall regression. For Cannonau, the inclusion of NHH and H_T > 35 °C_ as predictor variables (model 2) improved considerably the modeling performances as compared to the simple linear model. Both variables were highly significant correlated to TSA and increased regression significance, R, R^2^ and Adjusted R^2^ (Adj.R^2^), respectively to: 0.0001, 0.649, 0.421 and 0.378. The third model is divided into two alternatives, using: (a) H_T < 17 °C_ and (b) H_T < 15 °C_. The model including the three independent variables, NHH, H_T > 35 °C_ and H_T < 17 °C_ (model 3), was the one that explained the most of the variations in Bovale Grande TSA, about 75.9%, without showing collinearity problems. Also in Cannonau, this model accounted for a much higher proportion of total anthocyanin variation than the previous two, although total berry skin anthocyanin contents still remained quite weakly associated with the temperature driving variables (Adj.R^2^ = 0.419).

In the last model, after adding NHH, the β coefficient of the variable representing prevalence of low temperatures assumed negative sign in the correlation with TSA in Bovale Grande. On the contrary, in Cannonau the permanence of low temperatures (H_T < 17 °C_ or H_T < 15 °C_) have demonstrated a positive role in TSA accumulation model 3 while H_T > 35 °C_ assumed negative influence, meaning that holding constant the other predictors, a variation of +1 in H_T > 35 °C_ results in a reduction of −0.209 in TSA.

**Table 5:** General model estimates of Bovale Grande berry TSA (mg malvidin kg^−1^), linear regression analysis and model performance.

Model	Predictors	Descriptive Statistics			Model Performance									
		N	df_1_	df_2_	Regression Sig.	R	R^2^	Adj. R^2^	Unstandardized Coefficients		Variables Significance		Collinearity Statistics	
									β	Std. Error	T	Sig.	Tolerance	VIF
**1**	**Intercept**	30	1	28	0.0001	0.861	0.741	0.732	50.348	29.272	1.72	0.096		
	**NHH**								0.594	0.066	8.845	0.000	1	1
**2**	**Intercept**	30	1	27	0.0001	0.865	0.748	0.730	44.92	29.988	1.498	0.146		
	**NHH**								0.552	0.081	6.79	0.000	0.671	1.489
	**H_T > 35 °C_**								0.41	0.456	0.899	0.377	0.671	1.489
**3**	**Intercept**	30	1	26	0.0001				43.206	29.741	1.524	0.140		
	**NHH**								0.808	0.153	5.538	0.000	0.187	5.359
	**H_T > 35 °C_**								0.108	0.478	0.238	0.814	0.602	1.661
	**H_T < 17 °C _^(a)^**					0.887	0.784	0.759	−1.084	0.527	−2.059	0.050	0.244	4.106
	**H_T < 15 °C _^(b)^**					0.873	0.762	0.735	−1.661	1.334	−1.245	0.224	0.288	3.473

Notes: **^(a)^** Model 3 performance using the predictors NHH, HT > 35 °C and HT < 17 °C; **^(b)^** Model 3 performance using the predictors NHH, HT > 35 °C and HT < 15 °C.

**Table 6:** General model estimates of Cannonau berry TSA (mg malvidin kg^−1^), linear regression analysis and model performance.

Model	Predictors	Descriptive Statistics			Model Performance									
		N	df_1_	df_2_	Regression Sig.	R	R^2^	Adj. R^2^	Unstandardized Coefficients		Variables Significance		Collinearity Statistics	
									β	Std. Error	T	Sig.	Tolerance	VIF
**1**	**Intercept**	30	1	28	0.004	0.511	0.261	0.235	71.013	7.776	9.133	0.000		
	**NHH**								0.055	0.017	3.145	0.004	1	1
**2**	**Intercept**	30	1	27	0.001	0.649	0.421	0.378	75.066	7.168	10.473	0.000		
	**NHH**								0.083	0.019	4.412	0.000	0.698	1.432
	**H_T > 35 °C_**								−0.271	0.099	−2.727	0.011	0.698	1.432
**3**	**Intercept**	30	1	26	0.001				76.575	6.983	10.966	0.000		
	**NHH**								0.035	0.033	1.052	0.302	0.207	4.840
	**H_T > 35 °C_**								−0.209	0.103	−2.036	0.052	0.611	1.637
	**H_T < 17 °C _^(a)^**					0.692	0.479	0.419	0.235	0.138	1.706	0.100	0.271	3.695
	**H_T < 15 °C _^(b)^**					0.681	0.463	0.401	0.42	0.292	1.44	0.162	0.434	2.306

Notes: **^(a)^** Model 3 performance using the predictors NHH, HT > 35 °C and HT < 17 °C; **^(b)^** Model 3 performance using the predictors NHH, HT > 35 °C and HT < 15 °C.

**Figure 5:**
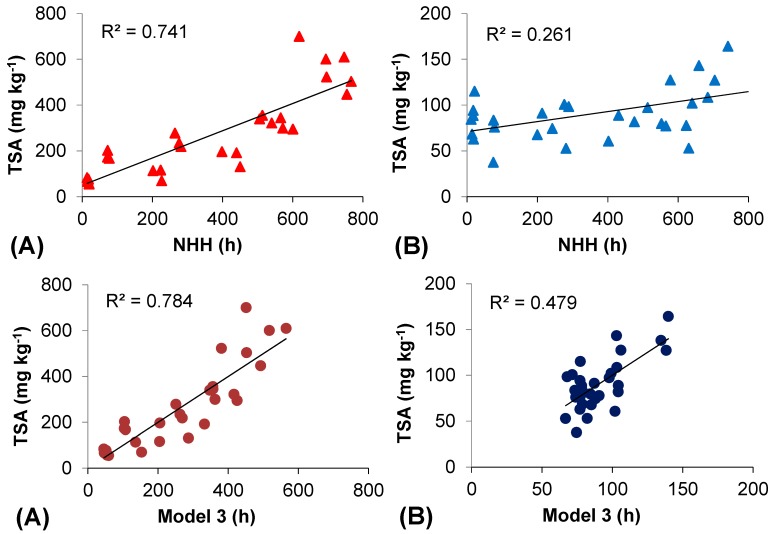
Relationships between total berry skin anthocyanin (TSA) content and the Normal Heat Hours (NHH) and between TSA and the regression model 3 in (**A**) Bovale Grande and (**B**) Cannonau datasets.

For both varieties, the introduction of H_T < 15 °C_ instead of H_T < 17 °C_ produced a smaller improvement on the regression models 1 and 2, reducing correlation and determination coefficients as compared to model 3(a) and adding no statistical significance to the prediction. In Figure 5, the relationships between the total berry skin anthocyanin and the NHH and the linear regression model 3(a), for Bovale Grande (A) and Cannonau (B) datasets, are represented. In both varieties, the addition of prevalence of high (T > 35 °C) and low (T < 17 °C) temperatures during ripening as predictors of TSA increased the model efficiency goodness in the linear regression, almost doubling the R^2^ for the Cannonau dataset.

A regression considering only NHH and H_T < 17 °C_, excluding H_T > 35 °C_, would not improve much the model performance in Bovale Grande (R = 0.885; R^2^ = 0.783, Adj.R^2^ = 0.767) and in Cannonau (R = 0.629; R^2^ = 0.396, Adj.R^2^ = 0.351) as compared to the NHH model. Despite the influence of other factors on anthocyanin accumulation in berry skin, the permanence of high and low temperatures can help explain the TSA accumulation pattern in Cannonau berries under warm climate conditions. In fact, when modeling TSA based on NHH and the permanence of temperatures higher than 35 °C and lower than 17 °C (Table 6) we observed much better fitting and model performance for Cannonau data than using only the NHH. Additionally, our results showed that the NHH model alone already represents a good estimator of TSA for Bovale Grande, also under warm climate conditions (Table 5).

## 3. Experimental Section

### 3.1. Plant Material and Experimental Site

During 2009, 2010 and 2011 growing seasons, an experimental trial was conducted in the red grapevine clonal collection of the University of Sassari, in Oristano, Italy (39°54'N, 8°37'E). In this field, the three-year-old Bovale Grande and Cannonau vines, grafted on 779 rootstock and spaced 2.5 m × 1.0 m, had North-South row orientation and were trained to a vertical trellis, Guyot pruned and drip-irrigated. From fruit set until harvest, three randomized blocks, with the two grapevine varieties arranged in two adjacent rows, were set covering 16 contiguous plants of each variety per block with two plastic films (UVA diffused and UV-A AV diffused, 1.5 mm thickness, Ginegar, Plastic Products, Ginegar, Israel) of different sunlight transmittance: visible + UV-A (Vis + UV-A) and visible (Vis). In every block, the UV-screening treatments were compared to 8 vines directly exposed to natural sunlight (Control). Each plastic tunnel entirely covered vines canopy and were completely opened at north and south sides and left opened laterally at the trunk base level to allow for better air circulation inside the tunnel. Inside the tunnels average air temperature differed less than 1.2 °C from the external ambient. Light spectral properties of the plastic films were tested using both portable spectroradiometer (FieldSpec^®^3, range 350–1800 nm) and UV-A (315–400 nm) and UV-B (280–315 nm) single sensors coupled to a portable datalogger (Skye Instruments Ltd., Llandrindod Wells, UK) and a ceptometer (Sunscan SS1 and BF3, Delta T Devices Ltd., Cambridge, UK) for measuring PAR interception throughout the canopy and reference ambient PAR above the canopy, respectively.

### 3.2. Light Microclimate Conditions

PAR interception transversal profiles at the fruit zone were measured at solar midday using a portable ceptometer connected to a total and diffuse PAR and sunshine sensor (Sunscan SS1 and BF3, Delta T Devices Ltd.). The mean values of PAR attenuation into the canopy layers were express as percentage of ambient photon flux density outside the canopy (% reference PAR). For monitoring UV-A and UV-B intensity interception at the fruit zone the single sensors were placed close to the clusters, transversely to its vertical axis, facing the east and west sides of row. Both light microclimate measurements were taken at the same time, in 12 replicates per treatment, along the canopy wall, and under clear sky conditions.

### 3.3. Season Thermal Conditions

Table 1 shows the temperature conditions during the three experimental seasons, gathered from the closest weather station (Capo Frasca, 39°45'41''N, 8°28'01''E) [43,44]. Maximum, mean and minimum monthly average temperatures of the period from June to September 2009, 2010 and 2011, as well as the variation between the values recorded during these months and those of the 30-year long term average, are reported.

### 3.4. Berry Temperature Monitoring and Determinations

Berry skin temperature was monitored during ripening, in four replicates of east and west side clusters, using fine-wire copper-constantan thermocouples (GMR Strumenti, Florence, Italy) collected to four-channel dataloggers (Zeta-tec Co., Cambridge, UK) for data registration at a 10 min intervals. In order to quantify the thermal effect of solar radiation on anthocyanin accumulation, the prevalence of given ranges of temperature (15 °C, 17 °C, and 35 °C) along each ripening season was then calculated and the thermal time was computed using the normal heat hours (NHH) model [46,47], which have been proven to give a explanation of vine thermal requirements for anthocyanin synthesis and accumulation [47,49].

### 3.5. Berry Composition Analysis

For each treatment, cluster fractions were randomly collected in two-week sampling intervals, from veraison until harvest, and berry weight and composition (total soluble solids, pH, titratable acidity, total skin anthocyanin and phenols content) was analyzed following OIV methodologies [50]. Berry anthocyanin composition was determined in a sample of 50 berry skin extracts per replicate previously frozen using HPLC, and following Di Stefano and Cravero method [51] as described in Fernandes de Oliveira and Nieddu [39].

### 3.6. Statistical Analysis

Berry composition data analysis was carried out using one-way ANOVA and the least significant difference (LSD) test to compare means at *p*-value of 0.05, using SPSS 16.0 (SPSS Inc., Chicago, IL, USA). Multiple linear regression was performed in order to examine the influence of accumulated NHH and of the permanence (in hours) of given temperature ranges (<15 °C, 17 °C and > 35 °C) on anthocyanin accumulation pattern in the two studied varieties over the three-year data set. For NHH calculation, 35 °C maximum, 10 °C minimum and 26 °C optimal temperatures for anthocyanin synthesis were considered [47], while the definition of the abovementioned temperature ranges took into consideration the observed 10th and 90th percentiles of berry skin temperatures along the duration of ripening in the three experimental seasons. The four independent variables tested for building the models (NHH, H_T < 15 °C_, H_T < 17 °C_ and H_T > 35 °C_) generally have high correlation degrees and therefore were sequentially added using hierarchical regression analysis and multicollinearity diagnostic (using tolerance and variance inflation factor, VIF statistics). The variables were tested for normality, and t-test coefficients were used to determine single variables explanatory power as well as the combination of variables that accounted for higher proportion of observed variance in berry total skin anthocyanin (TSA).

## 4. Conclusions

This study has demonstrated that natural UV light intensities can have a positive influence on total anthocyanin contents and may favor berry metabolism toward the accumulation of higher proportion of dihydroxylated derivatives and the more color stable acylated forms. Natural UV light intensities did not induce differences in trisubtituted anthocyanins as reported by other studies [36,37] but a positive effect of UV was observed in cyanidin and peonidin derivatives in Bovale Grande Control and Vis + UV-A in 2009 and in Cannonau in 2010. Both UV light and high permanence of elevated temperatures induced increased acylation levels though the major effect of solar radiation was driven by high temperatures, which were able to alter the proportion of anthocyanin derivatives and the degree of acylation to a greater extent, promoting higher accumulation of acetyl- and coumaroylglucosides, namely of the delphinidin and petunidin derivatives.

The relationship between TSS and TSA was quite different in the two varieties, since for Cannonau the increases in TSS were not followed by increasing TSA as observed in Bovale Grande. Such behavior could partially be explained by a high sensitivity to the permanence of critical ranges of temperature. In fact, besides the higher between-years variation in TSA contents observed in Cannonau, also, the modeling exercise provided evidence for a greater sensitivity to high and low temperature of berry anthocyanin contents in Cannonau.

In this work, we proved that NHH is a very good estimator of TSA for Bovale Grande but not for Cannonau. A positive contribution of the permanence of elevated temperatures (T > 35 °C) to anthocyanin accumulation was highlighted by the regression analysis for both varieties, especially for Cannonau dataset. The permanence of low (T < 17 °C) improved slightly TSA estimation, mostly for Cannonau.

Overall, the Normal Heat Hours model can explain a great part of variation in anthocyanin patterns, but in warm climate conditions and for varieties highly sensitive to temperature like Cannonau, the prediction of total skin anthocyanin contents can benefit from the addiction of variables that take into account the permanence of high and low temperatures until berry maturation.

Increasing the knowledge on how berry total skin anthocyanin and composition respond to natural UV radiation and temperature in Mediterranean areas can help improve cultivation practices, namely those affecting canopy microclimate, in order to favor the production of higher quality grapes. Likewise, assessing varietal sensitivity to these abiotic factors and enhancing the accuracy of anthocyanin accumulation estimation models represent an important contribution to a better vineyard management, starting from the varietal choices up to the harvest decisions.
